# THE ROLE OF SOCIAL SUPPORT IN SLEEP HEALTH FOLLOWING SPOUSAL LOSS: A LONGITUDINAL STUDY

**DOI:** 10.1093/geroni/igae098.3529

**Published:** 2024-12-31

**Authors:** Kristin Calfee, Soomi Lee

**Affiliations:** The Pennsylvania State University, University Park, Pennsylvania, United States; The Pennsylvania State University, University Park, Pennsylvania, United States

## Abstract

The loss of a spouse is consistently rated as one of the most stressful life events. Both spousal bereavement and divorce have been associated with poorer physical and mental health, including worsened sleep. However, the impact of spousal loss on sleep health may be mitigated by robust social support networks. This study utilizes a multidimensional measure of sleep health to examine its association with spousal loss, considering potential moderation by social support. 2,462 adults (Mage=66yrs) from the Midlife in the United States study provided longitudinal data at 2004-2005 and 2013-2016, 277 of whom lost a spouse to death or divorce between the two timepoints. A composite score of sleep regularity, satisfaction, alertness, efficiency, and duration was used to measure sleep health (higher=better). Social support was measured using Affectual Solidarity (AS), capturing both support and strain. General linear regression adjusted for sociodemographic covariates and depression was used to examine the association between spousal loss and sleep health, and the interaction between spousal loss and AS. Spousal loss was not a predictor of sleep health at follow-up. However, there was significant moderation by AS, such that the loss of a spouse was associated with worse sleep health for those who experienced a decrease in AS since the spousal loss (Slope=0.19, SE=.09, p=.03), but not for those who experienced an increase or no change in AS. Findings suggest that spousal loss may not necessarily degrade sleep health when social support is stronger. Future analyses will examine these associations separately by bereavement vs. divorce.

